# Transportation of patients on extracorporeal membrane oxygenation: a tertiary medical center experience and systematic review of the literature

**DOI:** 10.1186/s13613-016-0232-7

**Published:** 2017-02-07

**Authors:** Pedro Vitale Mendes, Cesar de Albuquerque Gallo, Bruno Adler Maccagnan Pinheiro Besen, Adriana Sayuri Hirota, Raquel de Oliveira Nardi, Edzangela Vasconcelos dos Santos, Ho Yeh Li, Daniel Joelsons, Eduardo Leite Vieira Costa, Flavia Krepel Foronda, Luciano Cesar Pontes Azevedo, Marcelo Park

**Affiliations:** 1 Intensive Care Unit, Hospital das Clinicas, University of São Paulo School of Medicine, Rua Dr. Enéas Carvalho de Aguiar, 255, Sala 5023, São Paulo, SP 05403000 Brazil; 2 Research and Education Institute, Hospital Sírio-Libanês, São Paulo, Brazil

**Keywords:** Acute respiratory failure, Extracorporeal membrane oxygenation, Transport, Rescue work

## Abstract

**Background:**

Utilization of extracorporeal membrane oxygenation (ECMO) has increased worldwide, but its use remains restricted to severely ill patients, and few referral centers are properly structured to offer this support. Inter-hospital transfer of patients on ECMO support can be life-threatening. In this study, we report a single-center experience and a systematic review of the available published data on complications and mortality associated with ECMO transportation.

**Methods:**

We reported single-center data regarding complications and mortality associated with the transportation of patients on ECMO support. Additionally, we searched multiple databases for case series, observational studies, and randomized controlled trials regarding mortality of patients transferred on ECMO support. Results were analyzed independently for pediatric (under 12 years old) and adult populations. We pooled mortality rates using a random-effects model. Complications and transportation data were also described.

**Results:**

A total of 38 manuscripts, including our series, were included in the final analysis, totaling 1481 patients transported on ECMO support. A total of 951 patients survived to hospital discharge. The pooled survival rates for adult and pediatric patients were 62% (95% CI 57–68) and 68% (95% CI 60–75), respectively. Two deaths occurred during patient transportation. No other complication resulting in adverse outcome was reported.

**Conclusion:**

Using the available pooled data, we found that patient transfer to a referral institution while on ECMO support seems to be safe and adds no significant risk of mortality to ECMO patients.

**Electronic supplementary material:**

The online version of this article (doi:10.1186/s13613-016-0232-7) contains supplementary material, which is available to authorized users.

## Introduction

Extracorporeal membrane oxygenation (ECMO) respiratory support is a potentially lifesaving strategy that has been shown to be cost-effective in high-income countries [1–3] and in a middle-income country [4]. Although its use remains restricted to cases of severe respiratory failure refractory to mechanical ventilation [5–8], further acute and chronic organ failures are very common and increase mortality and morbidity in these patients [9, 10]. Consequently, ECMO respiratory support is lifesaving only in highly select cases—namely, early after the onset of severe respiratory failure, and only in patients without severe acute or chronic organ dysfunctions [3, 5–7, 11, 12].

Facing the paucity of such cases, and the high level of expertise needed to implement ECMO support, a proper referral system becomes necessary and has been shown to be effective [7]. In the CESAR trial, the cost-effectiveness of ECMO respiratory support was demonstrated with 81% (73 out of 90) of ECMO-supported patients being transported to only one UK ECMO referral center [3]. However, the severity of those patients’ respiratory insufficiency makes transportation without ECMO support unsafe. Therefore, many referral centers transfer patients under ECMO respiratory support [13, 14] and, so far, there are no data regarding the safety of those inter-hospital ECMO transportations.

Considering the recent global increase in ECMO support, and the consequent increase in ECMO patient transportation, our objectives were the following: (a) to describe a Brazilian tertiary medical center’s experience with ECMO transportation of patients with severe respiratory failure, and (b) to describe the current literature by providing pooled results of mortality and complications associated with transportation on venovenous and venoarterial ECMO support.

## Background

### Patients of the Brazilian tertiary medical center

Patients’ data were retrieved from a prospectively collected REDCap database from the Hospital das Clínicas da Universidade de São Paulo [15]. From 2010 to 2014, physicians from various institutions directly contacted the Hospital das Clínicas ECMO team in cases of refractory hypoxemia. Initial evaluation was done through telephone contact together with a REDCap-based online form based on patients’ clinical and laboratory data [15]. The decision of whether or not to undertake the rescue was based on predefined inclusion and exclusion criteria. In cases of uncertainty, we required the consultation and approval of two other members of the ECMO team. The inclusion and exclusion criteria, as well as the clinical approach to support and ECMO initiation, were standardized and already published elsewhere [11, 16].

In all cases, cannulation was performed at bedside by the ECMO team at the consultant hospital. The femoral-to-internal jugular venovenous configuration was used. Bidimensional ultrasonography was used to guide the vascular puncture, guidewire insertion and cannulae positioning. A centrifugal magnetic pump with a Permanent Life Support (PLS) system using a polymethylpentene oxygenator (Rotaflow/Jostra Quadrox - D, Maquet CardiopulmonaryAG, Hirrlingen, Germany) was employed in all cases. Approximately 1 h after ECMO initiation, patients were transported in extracorporeal support to the Hospital das Clínicas da Universidade de São Paulo through ground ambulance or helicopter. The ECMO team for patient transportation consisted of 2 ICU physicians, 1 ICU nurse and 1 ICU respiratory therapist.

### Systematic review of the literature

#### Registration

This systematic review, including its search protocol, was registered on the PROSPERO database (registration number CRD42015024710).

#### Search strategy

We searched PUBMED (1966 until November 2012), EMBASE (January 1990 until November 2012), LILACS, and SCIELO to identify studies describing transportation of severely ill patients on ECMO support. Venovenous and venoarterial ECMO descriptions for respiratory support were included to better describe the population submitted to transportation while on ECMO. We relied upon observational studies, as no randomized studies comparing inter-hospital transportation of ECMO-supported patients have been published to date.

In order to enhance the sensitivity of our search, the terms were separated into two blocks for PUBMED, using medical subject headings [MeSH] and [All field] terms. The MeSH terms used in PUBMED were organized as follows: (“extracorporeal membrane oxygenation” or “extracorporeal oxygenation” or “extracorporeal life support” or ECMO) and (“transport” or “rescue work”). In the PUBMED, EMBASE, LILACS and SCIELO searches, the terms were inserted as [All fields] and were organized as follows: (“extracorporeal membrane oxygenation” or “extracorporeal oxygenation” or “extracorporeal life support” or ECMO) and (“transport” or “rescue work” or “rescue” or “retrieval”).

The resulting outputs were then combined. Duplicated results were excluded. The remaining articles were independently evaluated by 3 investigators (PVM, CAG and MP) for eligibility. Only manuscripts with the agreement of a least two investigators were included. We also searched personal records and the references of the retrieved articles for other potential studies.

#### Study evaluation and data extraction

Manuscripts were included only if data on patients’ transportation on ECMO support and hospital survival were available. Manuscripts involving animal data, case reports describing less than four subjects, language other than English, French, Spanish, or Portuguese and review articles were excluded. When available, the following data were also extracted: patients’ demographic features (including illness severity), mechanical ventilation information, complications during ECMO transportation, ECMO support data, and intensive care unit (ICU) and hospital lengths of stay. Retrieved data were also analyzed separately for pediatric patients and adult patients. When additional information was needed, an e-mail was sent to the main author requesting the data.

#### Quality assessment

Though there is no proper scoring system for evaluating case series of patients on ECMO support, we developed a predetermined system for all included studies based on previously published reports [17], and on commonly expected measures of quality for ECMO support and ECMO transportation. Each study was scored and a final grade was calculated to estimate data quality (Additional file). The studies’ final scores were classified by quality into three tertiles and mortality rate was assessed independently for each one of these groups.

#### Statistical analysis

Variables are shown as mean ± standard deviation if normally distributed, and median plus the 25th and 75th percentiles if otherwise. For descriptive data, a confidence interval was generated for each manuscript in accordance with the recommendations of the Association of Public Health Observatories (APHO) of England [18]. Some manuscripts did not present means and standard deviations for quantitative data. In these cases, means and standard deviations were estimated from the sample size, median, range or 25th and 75th percentiles, according to the Wan method [19]. Heterogeneity between and within studies was assessed using Cochran’s Q statistic and Higgin’s *I* ^2^. Either a *P* < 0.10 or an *I* ^2^ > 50% was considered suggestive of significant heterogeneity. We a priori expected that studies would present high heterogeneity. Therefore, we performed a random-effects meta-analysis using the DerSimonian and Laird method for all pooled characteristics and effects. The analyses were done using the metafor package from R open-source software [20].

## Results

### Brazilian medical center

During the studied period, our group was called on for consultation for a total of 12 patients. Seven patients fulfilled the predefined criteria and were supported by ECMO. All patients had received at least one rescue maneuver for refractory hypoxemia before ECMO initiation, as described in Table 1. In all cases, patients were cannulated at the remote institution and transferred under ECMO support to Hospital das Clínicas. Data on respiratory failure etiology, laboratory data, and ECMO settings are described in Table 1. Five patients were successfully weaned from ECMO support. One patient died 21 days after ECMO removal due to septic shock. Median time on ECMO support was 5 days [2, 10]. ICU and Hospital length of stay were 13 [2, 20] and 20 days [2, 60], respectively. Four patients survived to hospital discharge and were still alive after 90 days, with a survival rate of 57%.

**Table 1:** General characteristics, ICU support and diagnosis of the patients

General characteristics	All patients (*n* = 7)
Age (yo)	28 [14,48]
Male [*n*(%)]	3 [43]
SAPS 3	84 [57,118]
SOFA	13 [7,18]
Lung injury score	4 [3,4]
*Acute respiratory failure etiology*	
Pneumonia [*n*(%)]	4 [57]
Syncytial respiratory virus [*n* (%)]	1 [14]
Influenza A virus (H_3_N_2_) [*n* (%)]	1 [14]
Varicella zoster virus [*n* (%)]	1 [14]
Nosocomial pneumonia [*n* (%)]	2 [28]
Alveolar hemorrhage [*n* (%)]	1 [14]
*Pre*-*ECMO initiation hospital data*	
Hospital LOS (days)	8 [3,30]
Days on mechanical ventilation	8 [1,19]
*Pre*-*ECMO rescue maneuvers*	
Alveolar recruitment [*n* (%)]	7 (100)
Nitric oxide [*n* (%)]	1 [14]
Neuromuscular blockers [*n* (%)]	4 [57]
Corticosteroids [*n* (%)]	3 [43]
Tracheal gas insufflation [*n* (%)]	1 [14]
*Clinical outcomes*	
ECMO weaning [*n* (%)]	5 (71)
Time on ECMO support (days)	5 [2, 10]
Hospital discharge	4 (57)
90-day survival [*n* (%)]	4 (57)

Ventilatory settings	Pre-ECMO initiation	After ECMO initiation
Ventilatory mode (n)	6 PCV/1 VCV	7 PCV
Tidal volume (mL/kg)	310 [180, 500]	100 [12, 220]
Plateau pressure (cmH_2_O)	35 [31, 46]	20 [20, 25]
PEEP (cmH_2_O)	18 [13, 25]	10 [10, 20]
Resp. rate (insp./min.)	26 [25, 35]	10 [10, 10]
FiO_2_	1.0 [1.0, 1.0]	0.3 [0.21, 1.0]

Quantitative data are expressed as median [minimum, maximum]

*ECMO* extracorporeal membrane oxygenation, *ICU* intensive care unit, *SAPS 3* simplified acute physiological score, *SOFA* sequential organ failure assessment; the lung injury score is the Murray’s score; and the two patients in the puerperal period were, respectively, in the 1st and 10th day after delivery; *LOS* length of stay, *SBE* standard base excess, *PCV* pressure-controlled ventilation, *VCV* volume-controlled ventilation, *PEEP* positive end-expiratory pressure

Individual characteristics of the Brazilian patients transported on ECMO support are described in Additional file 1: Table 1S. The median transfer distance was 31 km [1,163] with an average rescue mission time of 345 min [270,960]. In one case, an electrical failure of the pump battery caused an unexpected pump arrest and the need for manual rotation of the hand crank during ground ambulance transportation. No deaths or any severe ECMO complications occurred during transport to our institution.

### Systematic review

#### Study inclusion

Our literature search yielded 610 publications for possible inclusion. Of these, 46 were excluded for duplicity. Of the remaining 564, 512 did not have data for hospital survival. Five studies required author contact for data clarification. Four authors responded to our solicitation and were included in the dataset. In the end, a total of 37 manuscripts were included for analysis (Additional file 2: Fig. 1S) [13, 14, 21–55]. Three studies were considered to be low quality, and 34 were considered moderate to high quality by our scoring system (Additional file 3: Table 3S). Our case series was included in the final analysis, resulting in a total of 38 manuscripts.

#### Outcomes

**Fig. 1:**
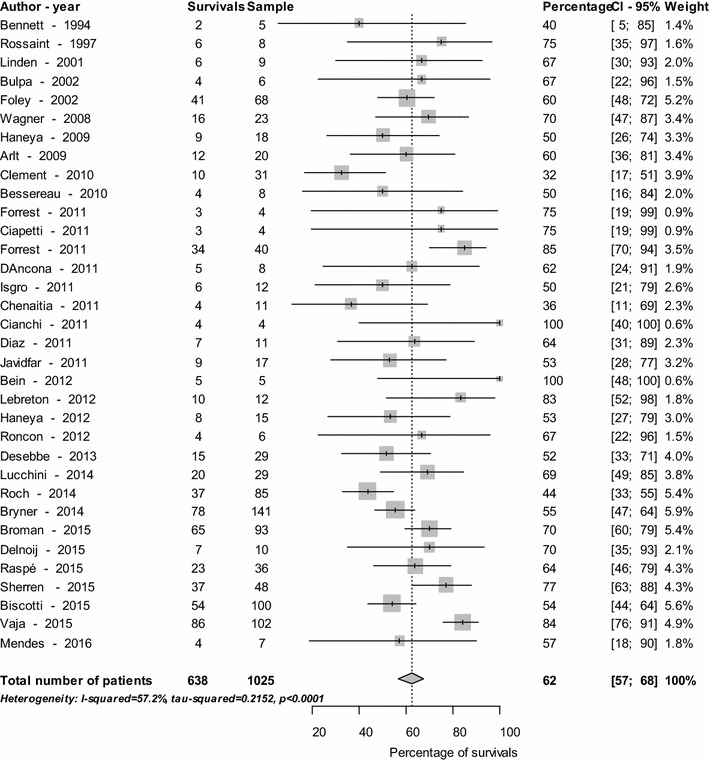
Forest plot of the adult case series of inter-hospital transportation on ECMO respiratory support, showing the weighted mean of hospital survival

**Fig. 2:**
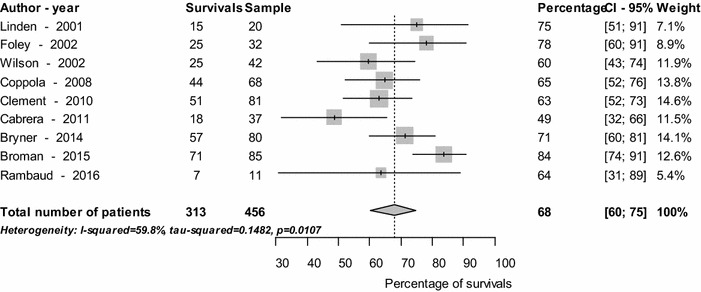
Forest plot of the pediatric case series of inter-hospital transportation on ECMO respiratory support showing the weighted mean of hospital survival

Mortality was assessed in all included studies for a total of 1481 patients. Of these, 1025 were adults, and 456 were pediatric. A total of 951 patients survived to hospital discharge after ECMO support in a crude analysis. The pooled survival rates for adult and pediatric patients were 62% (95% CI 57–68) and 68% (95% CI 60–75), respectively (Figs. 1, 2). After stratifying studies into tertiles of quality, we found a lower mortality rate in high-quality studies and a progressive increase in mortality for intermediary and lower tertiles. Mortality rates were 69% (95% CI 52–85%), 65% (95% CI 60–71%) and 61% (95% CI 46–76%) for the first, second and third tertiles, respectively. Total number of patients transported in each country is shown in Additional file 4: Figure 2s

General characteristics, pre-ECMO status, diagnosis and ECMO support data of the entire cohort are reported in Table 2. Most studies did not report any scoring system for hospital survival. Mean time on mechanical ventilation before ECMO initiation was 4.6 days (95% CI 3.7–5.5). Acute Respiratory Distress Syndrome was the most prevalent diagnosis reported for adult patients, while meconium aspiration syndrome was the diagnosis most frequently reported for the pediatric population. ECMO support duration, ICU length of stay and hospital length of stay were 9.8 (95% CI 8.6–10.9), 22 (95% CI 15–30) and 32 (95% CI 25–28) days, respectively.

**Table 2:** General characteristics, pre-ECMO status, diagnosis and ECMO support data of the patients

Characteristic	≥12 years old		<12 years old	
	Patients analyzed Total *N* = 682	Mean (CI 95%)	Patients analyzed Total *N* = 365	Mean (CI 95%)
Age (yo)	627	38 (35–41)	114	3.1 (1.4–4.8)
Male gender (%)	482	61 (45–77)	365	17 (13–21)
Weight (kg)	119	74 (67–82)	37	23.6 (17.6–29.6)
*Pre*-*ECMO status*				
Time on MV (days)	246	4.6 (3.7–5.5)	–	–
pH	242	7.24 (7.19–7.28)	32	7.26 (7.23–7.29)
P/F ratio (mmHg)	373	59 (40–78)	–	–
PCO_2_ (mmHg)	306	71 (63–78)	–	–
APACHE II	53	22 (21–23)	–	–
SAPS 2	128	46 (40–52)	–	–
SAPS 3	10	86 (80–92)	–	–
*Diagnosis*				
ARDS (%)	682	30 (26–34)	365	14 (11–18)
Bacterial pneumonia (%)	682	27 (23–31)	365	6 (4–9)
Viral pneumonia (%)	682	22 (19–25)	365	5 (3–7)
Respiratory and right ventricle failure (%)	682	19 (12–26)	365	17 (13–21)
Trauma (%)	682	4 (3–6)	365	0
Aspiration syndromes (%)	682	3 (1–4)	365	1.0 (0.1–2.5)
Meconium aspiration syndrome (%)	682	0	365	19 (15–23)
Congenital diaphragmatic hernia (%)	682	0	365	8 (5–11)
Persistent newborn pulmonary hypertension (%)	682	0	365	8 (5–10)
*ECMO support and outcome data*				
ECMO support duration (days)	458	9.8 (8.6–10.9)	7.5	–
Venovenous configuration (%)	682	62 (59–66)	365	42 (35–50)
Venoarterial configuration (%)	682	16 (13–19)	365	58 (50–65)
ICU LOS (days)	64	22 (15–30)	–	–
Hospital LOS (days)	119	32 (25–38)	–	–

*ECMO* extracorporeal membrane oxygenation, *CI* confidence interval, *BMI* body mass index, *MV* mechanical ventilation, *APACHE* Acute Physiological and Chronic Health Evaluation score, *SAPS* Simplified Physiological Score, *ARDS* acute respiratory distress syndrome, *ICU* intensive care unit, *LOS* length of stay

At least one complication was reported in 12 of the 38 manuscripts analyzed, totaling 80 occurrences in 1481 patients transported (Additional file 5: Table 2S). The most common complication was sudden fall in tidal volume during transportation. We found two reports of deaths during patient transportation to the referral institution. Rescue mission characteristics and adverse events are described in Table 3.

**Table 3:** Rescue mission characteristics and adverse events

	Patients analyzed (Total *N* = 1481)	Mean (CI 95%)
*Rescue mission*		
Distance of transportation (km)	444	486 (68–904)
Rescue time lasting (hours)	318	17 (9–27)
Ambulance (%)	1481	53 (50–56)
Fixed wing (%)	1481	27 (25–31)
Helicopter (%)	1481	22 (19–24)
International transportation (%)	1481	7 (5–8)
*Adverse events on the rescue mission*		
Death during transport (%)	1481	2 deaths (0.1%)
Tidal volume fall (%)	1481	4 (3–5)
System rupture (%)	1481	1.0 (0.4–1.6)
Any bleeding (%)	1481	0.9 (0.3–1.4)
Power failure (%)	1481	0.5 (0.1–0.9)
Hypotension (%)	1481	0.4 (0.1–0.8)
Cannulae dislodgement (%)	1481	0.2 (0.1–0.7)
Others (%)	1481	0.9 (0.3–1.4)
Absence of complications (%)	1481	92 (91–94)

## Discussion

ECMO remains a high-cost therapy with lifesaving potential in a select group of critically ill patients [3].Given the level of expertise needed for daily care of these patients, it is preferable that ECMO candidates be transferred to a specialized referral center. Because of the severe respiratory failure, patient transfer without ECMO is usually deemed to be too risky. Conversely, there are no data regarding the safety of inter-hospital ECMO transportations. Herein, we report a case series of patients transported under ECMO support to a referral hospital in Brazil with a survival rate of 57% and no major complications or deaths during transportation. Additionally, our systematic review of the literature showed a pooled survival rate for adult and pediatric patients of nearly two-thirds—with just 2 deaths reported in this cohort of 1481 patients—and without any other major adverse events resulting from the transportation itself.

Our data are compatible with the overall mortality reported in the latest publication by the Extracorporeal Life Support Organization (ELSO), in which the expected survival rate for adult venovenous extracorporeal support was 58% [56]. Similarly, using the available published data, we found a survival rate of 62% for adult patients transported while on ECMO. For the pediatric population, our pooled analysis retrieved a survival rate of 68%, in comparison with the 57% reported in ELSO guidelines [56]. Therefore, in this case series and in our overall analysis, we found no increase in mortality for ECMO support despite the need for patient transfer to a referral institution.

Concerns regarding the safety of transport of critically ill patients in need of extracorporeal support are an important question to be solved considering the recent global increase in ECMO support [5, 56, 57]. In the Cesar trial, patients who were randomized for the ECMO group were transferred to the referral center only after transport was considered safe by the ECMO Team, therefore delaying the initiation of support. As reported in the text, patients were not transported while on ECMO and, despite precautions, two deaths were reported during patient transfer [3]. Similarly, in a previous publication of 158 infants accepted for ECMO initiation, Boedy et al. reported 18 (39.1%) deaths associated with transport. Five infants died waiting for ECMO initiation and 13 died either during transport without ECMO assistance or, after arriving moribund, before ECMO could be started. Considering all these deaths occurred before ECMO initiation, the authors concluded that there may be a hidden mortality associated with ECMO transportation that is generally excluded when we look exclusively at ECMO-supported patients [58]. Therefore, a strategy of rapid ECMO initiation and patient transport while on ECMO support may be safer than the use of conventional mechanical ventilation during transfer to the referral center.

However, it is important to highlight that the presence of complications is common, and nearly a third of the analyzed studies reported at least one complication during transport. Sudden fall in tidal volume was the most common complication reported. Power failure, circuit rupture and other more severe complications were also reported, but no deaths or any adverse outcomes related to these complications during transport were described. It is interesting to note that the majority of complications (74%) were reported in one single study [43] (Additional file 5: Table 2s), suggesting that most reports on ECMO transportation did not focus on looking for adverse events. Certainly, the definition of complications during patient transportation varied between studies, making it difficult to understand the real size of this problem. It is very likely that the incidence of adverse events is much higher than described in this manuscript.

Our study has several limitations: (1) The absence of any scoring system in most of the studies makes it difficult to correlate expected mortality with final results. However, as previously described, overall mortality rate was compatible with the expected mortality previously published in the ELSO guidelines for ECMO-supported patients. (2) A publication bias may have affected our results. As observed in our analysis, low-quality studies were associated with a high mortality rate and, possibly, even higher mortality rates may be found in unpublished data. (3) No randomized clinical trial has directly evaluated the safety of transporting ECMO patients. Our data were extracted mainly from case series, and the results are limited by the inherent flaws of such studies. (4) The studies included in this manuscript span several years of ECMO transportation worldwide. Therefore, clinical and technical development, which may have influenced the presence of complications and death in ECMO patients throughout the years, are not addressed in this manuscript.

## Conclusions

ECMO support is a high-cost therapy restricted to highly specialized referral centers. The analysis of pooled data from available literature suggests that patient transfer to a referral institution while on ECMO support seems to be safe and to not increase mortality in ECMO-supported patients. This modality should possibly be preferred over transport of such patients under conventional ventilation exclusively.

## Additional files

**Additional file 1. Table 1S:** Brasilian patients transported on ECMO support in Hospital das Clínicas.

**Additional file 2. Figure 1S:** Flow diagram of the systematic review according to the PRISMA statement.

**Additional file 3. Table 3S:** Quality evaluation of the manuscripts retrieved in accordance with our predefined score.

**Additional file 4. Figure 2S:** Number of transports in each country of the systematic review.

**Additional file 5. Table 2S:** Characteristics of each study retrieved from the systematic analysis.

## Abbreviations

ECMO: extracorporeal membrane oxygenation
ICU: intensive care unit
