# Supplementary material for: Transportation of patients on extracorporeal membrane oxygenation: a tertiary medical center experience and systematic review of the literature
Source: Ann Intensive Care. 2017 Feb 7;7:14. doi: 10.1186/s13613-016-0232-7 (PMC5296266; doi:10.1186/s13613-016-0232-7)
Supplement: Supplementary file 1 — Additional file 1. Table 1S: Brasilian patients transported on ECMO support in Hospital das Clínicas. [file 13613_2016_232_MOESM1_ESM.docx]

| **Table 1s: Individual characteristics of the Brazilian patients transported on ECMO support** | | | | | | | | | | | | | | | | |
| --- | --- | --- | --- | --- | --- | --- | --- | --- | --- | --- | --- | --- | --- | --- | --- | --- |
|  | |  | |  | |  |  |  |  |  |  |  |  |  |  |  |
|  | **Gender** | | **Age**  **(yo)** | | **Weight**  **(kg)** | | **Height**  **(cm)** | **60 days**  **survival** | **Transport** | **Distance (km)** | **Time**  **(min)** | **SAPS3** | **SOFA** | **Pulmonary**  **diagnosis** | **LIS** | **ECMO**  **weaning** |
|  |  | |  | |  | |  |  |  |  |  |  |  |  |  |  |
|  | F | | 14 | | 48 | | 158 | Yes | Ambulance | 0.60 | 360 | 105 | 18 | Alveolar hemorrhage | 4,00 | Yes |
|  | M | | 27 | | 84 | | 185 | No | Ambulance | 31.2 | 300 | 118 | 18 | Pneumonia | 4,00 | No |
|  | F | | 48 | | 50 | | 160 | Yes | Rotary wing | 31.2 | 270 | 84 | 10 | Pneumonia | 3,00 | Yes |
|  | M | | 16 | | 84 | | 190 | No | Ambulance | 23.2 | 420 | 57 | 7 | Pneumonia | 3,00 | Yes |
|  | F | | 31 | | 55 | | 160 | No | Ambulance | 12.2 | 345 | 80 | 13 | H3N2 | 3,70 | No |
|  | M | | 31 | | 75 | | 185 | Yes | Ambulance | 163.0 | 960 | 84 | 13 | Varicella zoster | 4,00 | Yes |
|  | F | | 28 | | 75 | | 165 | Yes | Ambulance | 54.2 | 300 | 60 | 8 | RSV | 4,00 | Yes |
|  | |  | |  | |  |  |  |  |  |  |  |  |  |  |  |

ECMO denotes extracorporeal membrane oxygenation

LIS denotes lung injury score

SAPS denotes simplified acute physiological score

SOFA denotes sequential organ failure assessment

RSV denotes respiratory syncytial virus

H3N2 denotes Influenza A H3N2 virus infection
