# Supplementary material for: Transportation of patients on extracorporeal membrane oxygenation: a tertiary medical center experience and systematic review of the literature
Source: Ann Intensive Care. 2017 Feb 7;7:14. doi: 10.1186/s13613-016-0232-7 (PMC5296266; doi:10.1186/s13613-016-0232-7)
Supplement: Supplementary file 2 — Additional file 2. Figure 1S: Flow diagram of the systematic review according to the PRISMA statement. [file 13613_2016_232_MOESM2_ESM.docx]

**Other sources Records**

References of the articles 11

Personal communications 0

37 manuscripts included in the quantitative synthesis *

52 manuscripts assessed for eligibility

564 records screened

**Database Records**

PUBMED 585

SCIENCE DIRECT 7

LILACS 6

SCIELO 1

**Total 599**

46 duplicates removed

512 records excluded

15 manuscripts excluded:

Transportation without ECMO – 5

< 4 cases - 4

Only cardiac support - 2

Review manuscript – 2

Other language – 1

Non-answered email - 1

**Figure 1s:** Flow diagram of the systematic review according to the PRISMA statement.

* This manuscript was also included in the final analysis as the 38^th^ manuscript.
