# Supplementary material for: Transportation of patients on extracorporeal membrane oxygenation: a tertiary medical center experience and systematic review of the literature
Source: Ann Intensive Care. 2017 Feb 7;7:14. doi: 10.1186/s13613-016-0232-7 (PMC5296266; doi:10.1186/s13613-016-0232-7)
Supplement: Supplementary file 4 — Additional file 4. Figure 2S: Number of transports in each country of the systematic review. [file 13613_2016_232_MOESM4_ESM.docx]

**Figure 2s:** Number of patients’ transported on ECMO respiratory support per country up to 2015’s literature (Total of 1481 patients with 951 – 64% survivals).
