# Supplementary material for: Transportation of patients on extracorporeal membrane oxygenation: a tertiary medical center experience and systematic review of the literature
Source: Ann Intensive Care. 2017 Feb 7;7:14. doi: 10.1186/s13613-016-0232-7 (PMC5296266; doi:10.1186/s13613-016-0232-7)
Supplement: Supplementary file 5 — Additional file 5. Table 2S: Characteristics of each study retrieved from the systematic analysis. [file 13613_2016_232_MOESM5_ESM.docx]

| **Table 2s: Characteristics of the studies retrieved from the systematic analysis** | | | | | | | | | | | | |
| --- | --- | --- | --- | --- | --- | --- | --- | --- | --- | --- | --- | --- |
|  |  |  |  |  |  |  |  |  |  |  |  |  |
| **Pediatrics** | **Country** | **Year** | **International sample** | **Total sample** | **Survivals** | **Age**  **(years-old)** | **Mission time**  **(hours)** | **Mission distance (km)** | **P/F ratio**  **(mmHg)** | **pH** | **PaCO_2_**  **(mmHg)** | **Intercurrences**  **(occurrences)** |
|  |  |  |  |  |  |  |  |  |  |  |  |  |
| No | USA | 1994 | 0 | 5 | 2 | 33 | 48.4 | 141 | ---------- | ---------- | ---------- | None |
| No | Germany | 1997 | 0 | 8 | 6 | 34 | 8.55 | 495 | 42.9 | ---------- | 57.8 | None |
| No | Sweden | 2001 | 0 | 9 | 6 | 29 | 2.7 | 296 | 52 | ---------- | ---------- | Non-declared |
| No | Belgium | 2002 | 0 | 6 | 4 | > 12 | 40 | ---------- | 45 | ---------- | ---------- | 1 |
| No | USA | 2002 | 0 | 68 | 41 | 38 | 7.15 | 352 | ---------- | 7.29 | 49 | 6 |
| No | Norway | 2008 | 0 | 23 | 16 | 18 | ---------- | ---------- | ---------- | ---------- | ---------- | None |
| No | Germany | 2009 | 0 | 18 | 9 | ---------- | ---------- | 90 | ---------- | ---------- | ---------- | None |
| No | Germany | 2009 | 0 | 20 | 12 | 53 | ---------- | ---------- | 91 | ---------- | 50 | 1 |
| No | USA | 2010 | 0 | 31 | 10 | > 12 | ---------- | ---------- | ---------- | ---------- | ---------- | None |
| No | France | 2010 | 0 | 8 | 4 | 35 | 6.12 | ---------- | ---------- | ---------- | ---------- | Non-declared |
| No | Australia | 2011 | 4 | 4 | 3 | 31 | 19 | 2020 | 53 | 7.17 | 92 | 2 |
| No | Italy | 2011 | 0 | 4 | 3 | 42 | 4.75 | 178 | 49 | 7.26 | 67 | None |
| No | Australia | 2011 | 0 | 40 | 34 | 34 | 8.4 | 245 | 57 | 7.21 | 74 | None |
| No | Italy | 2011 | 0 | 8 | 5 | 38 | 10 | ---------- | ---------- | ---------- | ---------- | 1 |
| No | Italy | 2011 | 0 | 12 | 6 | 35 | 10 | 102 | 61 | 7.28 | 75.1 | None |
| No | France | 2011 | 0 | 11 | 4 | 38 | ---------- | 45 | ---------- | ---------- | ---------- | None |
| No | Italy | 2011 | 0 | 4 | 4 | 41 | ---------- | ---------- | 51 | 7.25 | 77 | None |
| No | Chile | 2011 | 0 | 11 | 7 | 32 | ---------- | ---------- | ---------- | ---------- | ---------- | None |
| No | USA | 2011 | 0 | 17 | 9 | 38 | 67.6 | 50.6 | 55 | ---------- | ---------- | None |
| No | Germany | 2012 | 5 | 5 | 5 | 23 | ---------- | 411 | 65 | 7.16 | 89 | None |
| No | France | 2012 | 3 | 12 | 10 | 37 | 2.1 | 901 | ---------- | ---------- | ---------- | Non-declared |
| No | Germany | 2012 | 0 | 15 | 8 | 48 | ---------- | ---------- | 60 | 7.24 | 75 | None |
| No | Portugal | 2012 | 0 | 6 | 4 | 41 | ---------- | ---------- | 70 | 7.33 | 62 | None |
| No | France | 2013 | 0 | 29 | 15 | 41 | 48 | 38 | 60 | ---------- | ---------- | None |
| No | Italy | 2014 | 0 | 29 | 20 | 40 | 9.3 | 132 | ---------- | 7.28 | 70.7 | 1 |
| No | France | 2014 | 0 | 85 | 37 | 47 | ---------- | ---------- | ---------- | ---------- | ---------- | None |
| No | USA | 2014 | 0 | 141 | 78 | 13 | 7.1 | 172 | ---------- | ---------- | ---------- | 1 |
| No | Sweden | 2015 | 53 | 93 | 65 | 49 | ---------- | ---------- | 59 | ---------- | ---------- | 59 |
| No | Netherlands | 2015 | 0 | 10 | 7 | 46 | ---------- | 92.8 | 90.8 | ---------- | ---------- | None |
| No | Germany | 2015 | 0 | 36 | 23 | 49 | ---------- | 52.3 | 92.3 | ---------- | 77 | Non-declared |
| No | UK | 2015 | 0 | 48 | 37 | 44 | ---------- | ---------- | 76 | 7.14 | 78.8 | None |
| No | USA | 2015 | 0 | 100 | 54 | 38 | ---------- | ---------- | 55 | 7.27 | 57.4 | 1 |
| No | UK | 2015 | 0 | 102 | 86 | 41 | ---------- | 275 | 69 | 7.17 | 74 | None |
| No * | Brazil | 2015 | 0 | 7 | 4 | 30 | 8 | 57 | 47 | 7.29 | 69 | 2 |
| Yes | Sweden | 2001 | 6 | 20 | 15 | 1 | 3 | 427 | ---------- | ---------- | ---------- | 1 |
| Yes | USA | 2002 | 0 | 32 | 25 | 4 | 7.15 | 352 | ---------- | 7.26 | 48 | None |
| Yes | USA | 2002 | 0 | 42 | 25 | < 12 | ---------- | ---------- | ---------- | ---------- | ---------- | None |
| Yes | USA | 2008 | 0 | 68 | 44 | ---------- | ---------- | 4130 | ---------- | ---------- | ---------- | 2 |
| Yes | USA | 2010 | 0 | 81 | 51 | < 12 | ---------- | ---------- | ---------- | ---------- | ---------- | None |
| Yes | USA | 2011 | 0 | 37 | 18 | 3 | ---------- | ---------- | ---------- | ---------- | ---------- | 2 |
| Yes | Sweden | 2015 | 0 | 25 | 23 | 5 | ---------- | ---------- | 59 | ---------- | ---------- | Non-declared |
| Yes | Sweden | 2015 | 0 | 60 | 48 | ---------- | ---------- | ---------- | ---------- | ---------- | ---------- | Non-declared |
| Yes | France | 2016 | 0 | 11 | 7 | ---------- | 8 | 117 | 65 | 7.01 | 77 | None |
|  |  |  |  |  |  |  |  |  |  |  |  |  |

* This manuscript.
